# 

**Published:** 1893-11-11

**Authors:** 


CONTENTS.

**Sir Andrew Clark** - - - 81

**On Modern Progress in Ophthalmic Medicine and Surgery.** By Robert Brudenell Carter, F.R.C.S. - - 83

**The Medical Work of the Local Government Board.**—IV.—Locality and Disease - - 84

**Modern Aspects of Pain in Medicine.** II. - - 85

**The Field of Science** - - 86

**Annotations.**—Secret Remedies and Doctors' Consciences—Is Pain Necessary?—Diphtheria and Flats - - 87

**Notes and News** - - 88

**Scraps and Gleanings** - - 96

**Editor's Letter Box** - - 96

**The Book World of Medicine and Science** - - vii

**Medical Vacancies and Appointments** - - ix

**The Hospital Clinic**—

The Straight Position in Injuries of the Elbow-Joint - - 89

The Treatment of Myxœdema - - 90

New Appliances and Things Medical—Carbolic Disinfectants—Glenfield's Disinfecting and Cleansing Fluid—Borax Preparations - - 92

New Drugs and Preparations —Borax and Bromides in Epilepsy (*continued*) - - 92

**The Institutional Workshop**—

Within the Hospitals—King's College Hospital - - 93

Hospital Construction—New Home for Incurables, Newcastle-on-Tyne - - 94

Practical Points—Cottage Hospital Construction - - 96

The Story of the Insane from Year to Year - - 96

EXTRA SUPPLEMENT.—THE NURSING MIRROR.

**News from the Nursing World.**—Our Christmas Competitions—Unwelcomed Frankness—Hospital and Asylum Workers—Heavy Cases—The Civil Hospital, St. Helena—Another New Home—A Praiseworthy Decision—A Toronto Association—Vanished Joys—Short Items - - li
**On the Nursing of Diseases of the Stomach.** I.—Introductory - - liii
**Where to Go,** liii; **Novelties for Nurses** - - lvii
**Nursing in the United States.**—Proper Organisation of Training Schools (*continued*). By Miss Darche - - liv
**Nursing in Newfoundland.**—Mission to Deep-Sea Fishermen—Labrador (*continued*) - - lv
**Nursing in Switzerland** - - lvi
**Belgrave Hospital for Children,** lvi; **Nurses at Worthing** lvii
**Everybody's Opinion.** — Children's Wards in Infectious Hospitals - - lvii
**For Reading to the Sick.**—Shaping the Stones - - lvii
**Notes and Queries** - - lvii
**Causerie from New England** - - lviii
**The Book World for Women and Nurses** - - lix

